# Metagenome analysis using serum extracellular vesicles identified distinct microbiota in asthmatics

**DOI:** 10.1038/s41598-020-72242-w

**Published:** 2020-09-15

**Authors:** Ji-Hyang Lee, Jun-Pyo Choi, Jinho Yang, Ha-Kyeong Won, Chan Sun Park, Woo-Jung Song, Hyouk-Soo Kwon, Tae-Bum Kim, Yoon-Keun Kim, Hae-Sim Park, You Sook Cho

**Affiliations:** 1 Division of Allergy and Clinical Immunology, Department of Internal Medicine, Asan Medical Center, University of Ulsan College of Medicine, 88 Olympic-ro 43-gil, Songpa-gu, Seoul, 05505 Republic of Korea; 2 Department of Internal Medicine, Seoul National University Bundang Hospital, Seongnam, Republic of Korea; 3 MD Healthcare Inc, Seoul, Republic of Korea; 4 Department of Internal Medicine, Veterans Health Service Medical Center, Seoul, Republic of Korea; 5 Department of Internal Medicine, Inje University Haeundae Paik Hospital, Busan, Republic of Korea; 6 Department of Allergy and Clinical Immunology, Ajou University Medical Center, Suwon, Republic of Korea

**Keywords:** Asthma, Diagnostic markers

## Abstract

Different patterns of bacterial communities have been reported in the airways and gastrointestinal tract of asthmatics when compared to healthy controls. However, the blood microbiome of asthmatics is yet to be investigated. Therefore, we aimed to determine whether a distinct serum microbiome is observed in asthmatics by metagenomic analysis of serum extracellular vesicles (EVs). We obtained serum from 190 adults with asthma and 260 healthy controls, from which EVs were isolated and analyzed. The bacterial composition of asthmatics was significantly different from that of healthy controls. Chao 1 index was significantly higher in the asthma group, while Shannon and Simpson indices were higher in the control group. At the phylum level, Bacteroidetes was more abundant in asthmatics, while Actinobacter, Verrucomicrobia, and Cyanobacteria were more abundant in healthy controls. At the genus level, 24 bacterial genera showed differences in relative abundance between asthmatics and controls, with linear discriminant analysis scores greater than 3. Further, in a diagnostic model based on these differences, a high predictive value with a sensitivity of 0.92 and a specificity of 0.93 was observed. In conclusion, we demonstrated distinct blood microbiome in asthma indicating the role of microbiome as a potential diagnostic marker of asthma.

## Introduction

Asthma is a heterogeneous and multifactorial disease of the airways. The role of the microbiome was first suggested as a contributor to the development of asthma, which aligned with the “hygiene hypothesis”. It has been postulated that early-life exposure to diverse microbiomes is a protective factor against the development of allergic diseases^1^. Unfortunately, the application of this hypothesis may be limited to early-onset asthma.

Historically, the lungs have been regarded as sterile without bacterial colonization^2^. Although the respiratory mucosa harbors a diverse community of microorganisms, traditional culture-dependent methods could only identify a limited number of bacterial species^3^. Recent advancements in sequencing technologies, however, have identified microbes that colonize the airways and lungs of healthy individuals and respiratory diseases^4^. Accordingly, distinct respiratory microbiota patterns in asthma patients have been identified compared to healthy controls^5^. Along with deeper understanding of heterogeneity underlying asthma, physicians have explored the relationships between respiratory microbiota and the features of asthma. Despite the differences in the reported compositions, different patterns of microbiota, such as bacterial and even fungal, were revealed, according to asthma characteristics^6,7^. These results suggest the potential of the microbiome as a determinant factor in asthma pathogenesis.

Dysbiosis of gut microbiota has long been shown to increase susceptibility to asthma. Accumulating evidence has shown the mechanisms of interaction between gastrointestinal and respiratory systems in both children and adults^8,9^. This so-called “gut–lung axis” highlights the importance of the gut microbiome in respiratory diseases.

However, it is still inconclusive which sampling compartment best represents the asthma status. Moreover, the different bacterial composition along the airway, from the nasal cavity to the lungs, complicates this issue^10^. Additionally, the absence of standardized methods for obtaining a specimens for microbiome analysis is another challenge in interpreting and comparing the results across different studies. There is also a dilemma in choosing the tools, since swabbing, bronchoscopy, and collecting sputum or stool all pose disadvantages such as contamination, invasiveness, and patient discomfort^10^.

As a culture-independent method, metagenomics using extracellular vesicles (EVs) has been suggested for 16 s rDNA sequencing to identify microbiomes. EVs are 20–200 nm-sized molecules secreted by bacteria. They contain genomic DNA fragments of bacteria and induce the host immune response^11^. Given their stability and distribution throughout the body, EVs might reflect the bodily microbiota of a host^12^. The usefulness of blood EVs for metagenomic analysis of microbiota has been reported in a murine model of Alzheimer’s disease (AD). Although AD is a brain disorder, blood EVs showed distinct microbiota in AD mice, which was explained, in part, by the gut-brain interaction^13^. Blood is easy to obtain and able to reflect systemic conditions; thus, it has potential to be a practical diagnostic tool. However, to our knowledge, there is no study investigating blood EVs for metagenomics in asthma.

In this study, we performed metagenomic analysis using blood EVs to determine whether a distinct microbiome is observed in asthma patients compared to healthy controls. In addition, we attempted to characterize the microbiota according to clinical characteristics of asthma. Based on the results of the microbiota composition between asthmatic and healthy controls, we suggested a diagnostic model of asthma.

## Results

### Characteristics of study subjects

A total of 190 adults with asthma were included for analysis. The clinical characteristics of the study subjects are summarized in Table 1. The healthy control group was composed of 155 females and 105 males with a mean age of 56 years. The concentrations of genomic DNA were 0.81 ± 2.19 µg/µL in healthy controls and 0.59 ± 1.63 µg/µL in asthmatics.

**Table 1:** Baseline characteristics of asthmatics.

Characteristics	Asthmatics
Age, mean ± SD	48.8 ± 14.6
Females, n (%)	114 (60)
BMI, mean ± SD	24.1 ± 3.6
**Atopy status**	
Atopic, n (%)	49 (25.8)
Non-atopic, n (%)	62 (32.6)
Unknown, n (%)	79 (41.6)
**Blood eosinophil counts**	
≥ 300/µL, n (%)	89 (46.8)
< 300/µL, n (%)	101 (53.2)
**Inflammatory type**	
Eosinophilic	44 (23.2)
Neutrophilic	29 (15.3)
Paucigranulocytic	70 (36.8)
Mixed granulocytic	21 (11.1)
Unknown, n (%)	26 (13.7)
**FEV1, predicted**	
≥ 60%, n (%)	162 (85.3)
< 60%, n (%)	27 (14.2)
Unknown, n (%)	1(0.5)
**Asthma medication**	
Steroid naïve, n (%)	21 (11.1)
ICS, n (%)	156 (82.1)
ICS and OCS, n (%)	12 (6.3)
Unknown, n (%)	1 (0.5)

*SD* standard deviation, *BMI* body max index, Forced expiratory volume in 1 s, *ICS* inhaled corticosteroids, *OCS* oral corticosteroids.

### Biodiversity in samples

**Figure 1:**
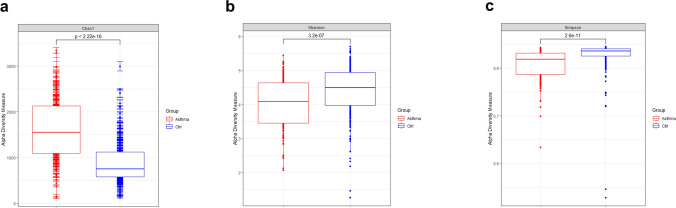
Comparison of biodiversity between asthmatics and healthy controls. Alpha diversity of blood bacteria in asthmatics (n = 260) and healthy controls (n = 190). The mean Chao1 index was 1596.5 in asthmatics and 887.9 in controls (**a**). The mean of Shannon index was 4.0 and 4.4 in asthmatics and controls, respectively (**b**). Lastly, the Simpson index was 0.9 in asthmatics and 1.0 in controls (**c**). The differences were statistically significant in all indices (*p* < 0.001).

Alpha-diversity was evaluated by Chao1, Shannon, and Simpson indices. The Chao1 index was significantly higher in the asthma group, while Shannon and Simpson indices were higher in the control group (Fig. 1).

### Composition of bacterial communities

**Figure 2:**
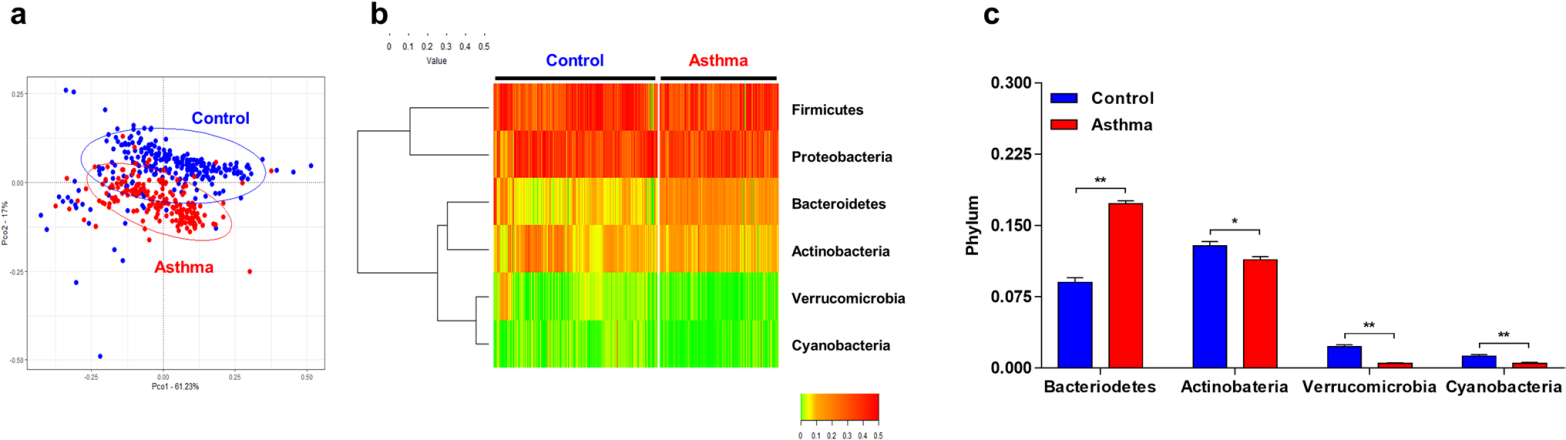
Comparison of bacterial communities at the phylum level in asthmatics and healthy controls. Principal coordinates analysis (PCoA) plot shows significantly different composition of microbial communities between asthmatics and heathy controls (*p* < 0.001) (**a**). The main distinct phyla in the serum of asthmatic patients and controls included Firmicutes, Proteobacteria, Bacteroidetes, Actinobacteria, Verrucomicrobia, and Cyanobacteria (**b**). Among these phyla, Bacteroidetes were more abundant in asthma patients, while Actinobacteria, Verrucomicrobia, and Cyanobacteria were more abundant in the controls (**c**).

**Figure 3:**
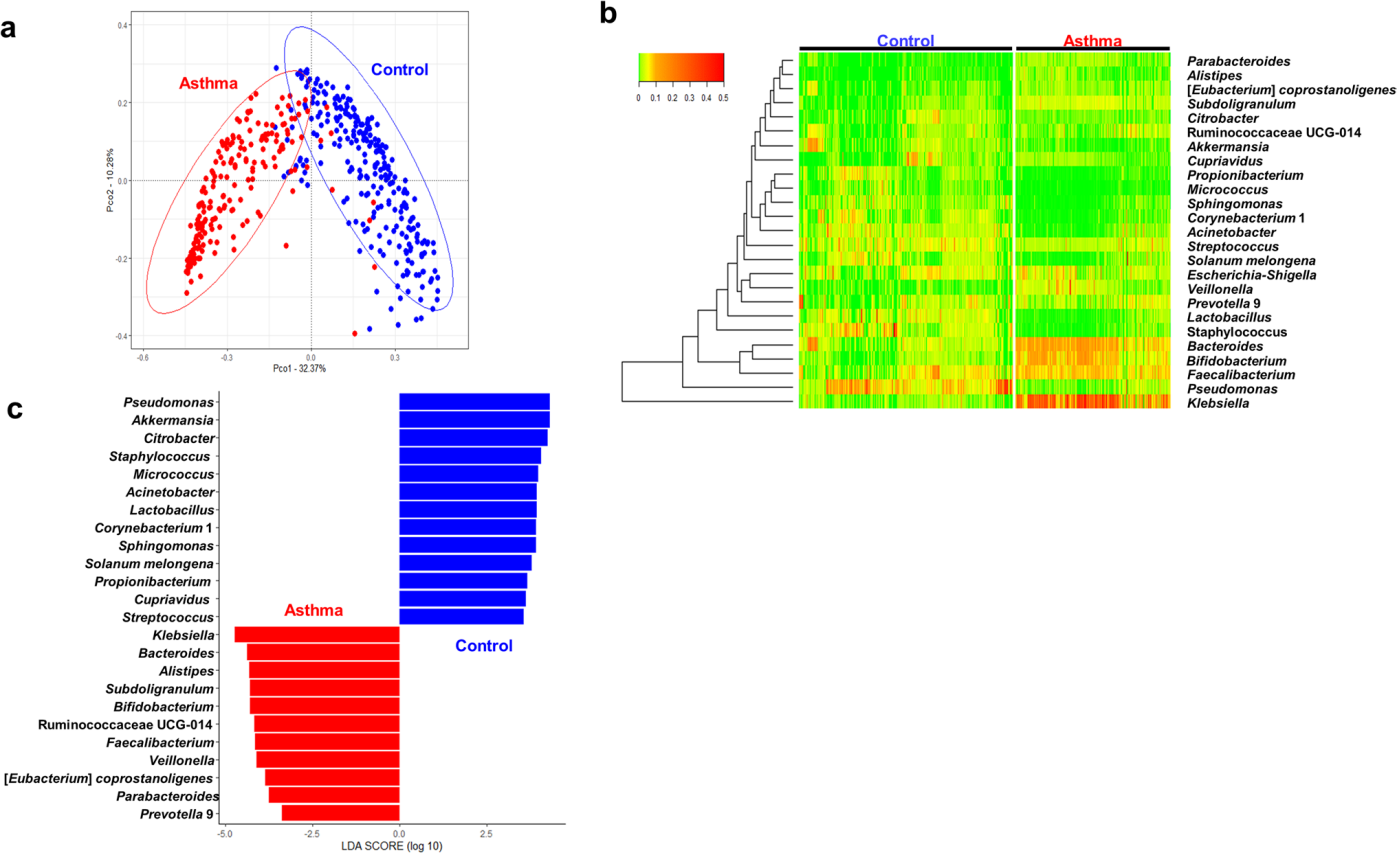
Comparison of bacterial communities at the genus level in asthmatics and healthy controls. At the genus level, the principal coordinates analysis (PCoA) plot also revealed discrete bacterial communities between the asthma and control groups (*p* < 0.001) (**a**). Heatmap shows relative abundance of the 25 major bacterial genera (**b**). A total of 24 bacterial genera were differently abundant between asthma patients and healthy controls, as defined by linear discriminant analysis scores greater than 3 (**c**).

At the phylum level, significant differences in the composition of microbial communities were found in asthmatic samples compared to heathy controls (*p* < 0.001) (Fig. 2a). The main distinct phyla in the serum of asthmatic patients and controls included Firmicutes, Proteobacteria, Bacteroidetes, Actinobacteria, Verrucomicrobia, and Cyanobacteria (Fig. 2b). Among these phyla, Bacteroidetes was more abundant in asthma patients, while Actinobacteria, Verrucomicrobia, and Cyanobacteria were more abundant in the controls (Fig. 2c). At the genus level, the PCoA plot also revealed discrete bacterial communities between the asthma and control groups (*p* < 0.001) (Fig. 3a). Figure 3b shows a heatmap of the relative abundance of the 25 major bacterial genera. When comparing relative abundances of bacteria, 24 bacterial genera showed differences in relative abundance between asthma patients and healthy controls with LDA scores > 3. Genera within the Bacteroidetes phylum, including *Bacteroides*,* Alistipes*,* Parabacteroides*, and *Prevotella*, were also significantly increased in asthmatics. Members of Proteobacteria phylum, including *Pseudomonas*,* Citrobacter*,* Acinetobacter*,* Sphingomonas*, and *Cupriavidus*, were notably abundant in healthy controls, whereas *Klebsiella* was more prevalent in asthmatics. Among the Firmicutes phylum, *Staphylococcus*,* Lactobacillus*, and *Streptococcus* showed a significant increase in healthy controls, while *Subdoligranulum*,* Ruminococcaceae*,* Faecalibacterium*,* Veillonella*, and *Eubacterium* were increased in asthmatics. Among the Actinobacteria phyla, *Micrococcus*,* Corynebacterium*, and *Propionibacterium* were more common in healthy controls, while *Bifidobacterium* was more common in asthmatics. *Akkermansia*, which belongs to Verrucomicrobia, was also notably abundant in the control group (Fig. 3c).

### Subgroup analysis according to asthma clinical features

**Figure 4:**
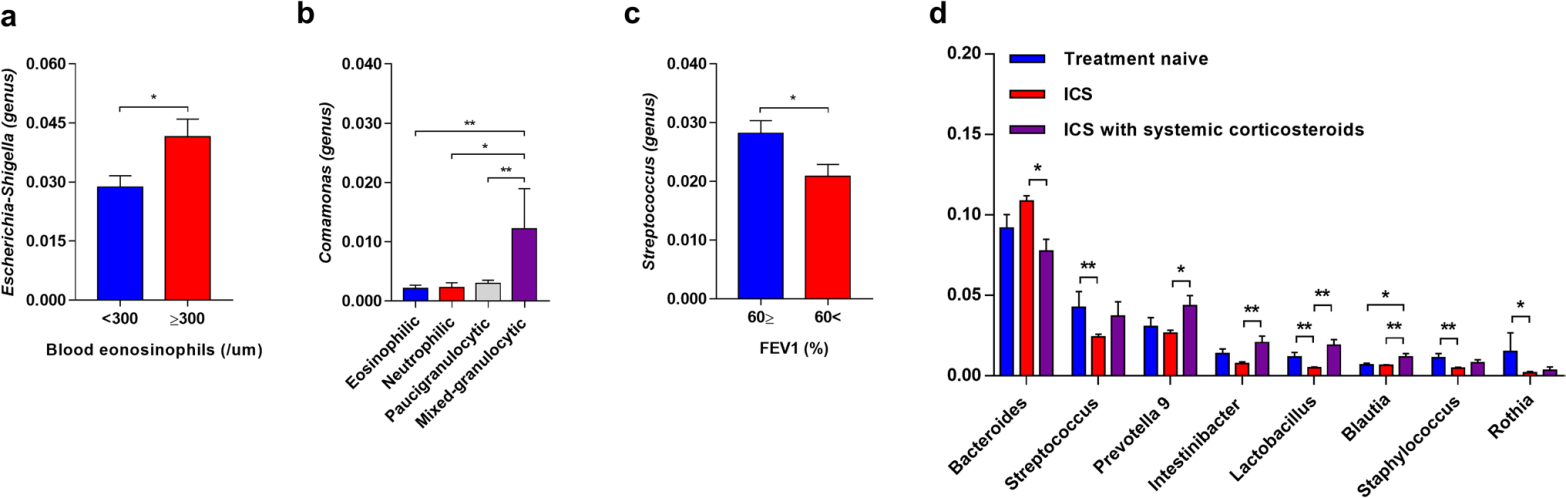
Subgroup analysis according to clinical features of asthma at the genus level. Subgroup analyses identified distinct serum microbiota according to blood eosinophil counts (**a**), inflammatory type of induced sputum (**b**), lung function (**c**), and medication (**d**) in patients with asthma.

To further explore the relationship between the microbiota composition and the clinical features of asthmatics, we classified the patients based on inflammation type and lung function. In a comparison between eosinophilic and non-eosinophilic asthma based on peripheral blood eosinophil count (with cutoff level of 300/µL), *Escherichia/Shigella* was the only bacteria that was more abundant in serum with higher eosinophils at the genus level (Fig. 4a). We also classified the patients into four inflammatory phenotypes according to the granulocytic cell count of their sputum, namely, eosinophilic, neutrophilic, paucigranulocytic, and mixed granulocytic asthma. Although the microbiome composition at the phylum level was comparable among the groups, *Comamonas* exhibited significant abundance in the mixed granulocytic asthma (Fig. 4b). When patients were grouped based on forced expiratory volume (FEV1) of 60%, *Streptococcus* was more enriched in patients with a higher lung function, FEV1 ≥ 60% (Fig. 4c). Finally, to assess the impact of corticosteroids on the microbiome, we classified patients based on corticosteroid usage (steroid naïve patients, patients using inhaled corticosteroids [ICS], and patients using oral corticosteroids [OCS]) (Fig. 4d). Regardless of corticosteroid use, no significant difference was noted at the phylum level. However, there were several distinct bacterial compositions at the genus level. In the steroid naïve patients, *Streptococcus*,* Rothia*,* Lactobacillus*, and *Staphylococcus* were more common than in patients using ICS. In ICS-treated patients, abundance of most bacteria was reduced, except *Bacteroides*. *Prevotella*,* Intestinibacter*,* Lactobacillus*, and *Blautia* were more common in patients using additional systemic corticosteroids than in patients using only ICS.

### Diagnostic model

**Figure 5:**
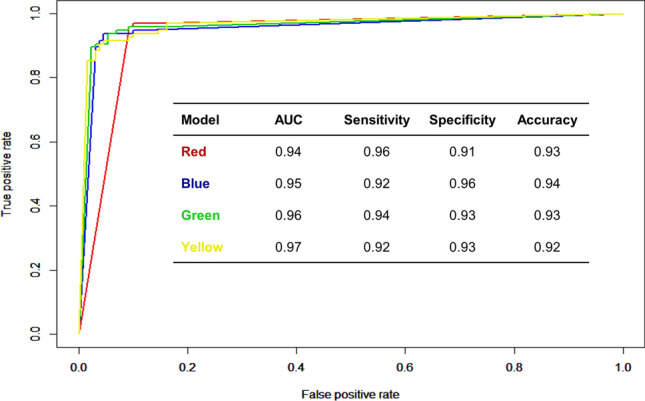
Diagnostic model of asthma based on distinct bacterial composition at the genus level. The diagnostic model using stepwise selection (red and blue) and linear discriminant analysis effect size (LEfSe) (green and yellow) reported high sensitivity and specificity. Red line, stepwise selection; blue line, stepwise selection with age and sex as covariates; green line, LEfSe; and yellow line, LEfSe with age and sex as covariates.

We proposed a diagnostic model based on metagenomic analysis. When using age and sex as covariates, the area under the curve for the diagnosis of asthma was 0.95 and 0.97 using stepwise selection (blue line; Fig. 5) and LEfSe (yellow line; Fig. 5), respectively.

## Discussion

This study demonstrated bacterial compositional differences at the phylum and genus levels between asthmatics and healthy controls using blood EVs. Patients with asthma showed significantly different richness and diversity indices compared to healthy controls. At the phylum level, Bacteroidetes was more abundant in asthmatics. Eleven bacterial genera, including *Klebsiella*,* Bacteroides*,* Alistipes*,* Subdoligranulum*, and *Bifidobacterium*, showed significant abundance in the serum EVs of asthmatic patients, whereas 13 genera, including *Pseudomonas*,* Akkermansia*,* Citrobacter*,* Staphylococcus*, and *Micrococcus*, were more abundant in controls. Additionally, a different pattern of serum microbiota was observed according to asthma clinical characteristics. Based on the distinct bacterial community, we suggested a diagnostic model of asthma with high sensitivity and specificity.

With regards to bacterial diversity in the asthmatic airway, inconsistent observations have been reported by other researchers. Even when using bronchial epithelial samples, some reported greater diversity in asthmatic samples, while others presented the opposite results. It was also noted that diversity varied significantly according to specimen type or sampling methods within individuals^14–16^. In analysis of bacterial-derived EVs, lower diversity was observed in asthmatics than healthy controls. Since we drew blood for analysis, our results may reflect the overall microbiota composition, unlike previous studies focusing on airways. Lower Shannon and higher Chao1 indices indicate that more diverse microbiota in asthmatics with highly uneven distributions. This alteration might be owing to the effects of corticosteroids providing opportunities for diverse bacteria to thrive in the human body.

Among dominant phyla known to reside in the lung, Firmicutes, Proteobacteria, Bacteroidetes, and Actinobacteria were also highly abundant in serum, suggesting that serum EVs can reflect bacterial composition of respiratory system to a substantial degree. When sera of asthmatics and healthy controls were compared, Bacteroidetes was the only phylum whose population was significantly increased in the asthma patients. (*p* < 0.01). Conversely, Actinobacteria, Verrucomicrobia, and Cyanobacteria were reduced in asthma patients. In a previous study analyzing the sputum from adult asthmatics, a decreased prevalence of Bacteroidetes was reported^17^. However, when using samples obtaining by left upper lobe brushing of adult asthmatics, increased prevalence of Bacteroidetes was observed^18^. Proteobacteria has been reported at a higher proportion in asthmatics than in healthy controls, which showed similar abundance in our study^19^. Thus, these inconsistencies in results may be largely due to differences in sampling sites, specimen collection methods, and participant characteristics.

The intestinal microbiome comprises the largest proportion of bacteria in the human body^20^. Further, a link between dysbiosis of the gut microbiome and various systemic diseases has been reported^21^. Phyla of Firmicutes, Actinobacteria, Bacteroidetes, Tenericutes, and Proteobacteria were reported as main members of the human gut microbiota^22^. Therefore, the serum microbiome in this study might represent a sum of bacterial communities from both pulmonary and gastrointestinal systems, considering the similarities of the compositions.

Several explicable mechanisms have been reported that demonstrate the effect of the gastrointestinal microbiome on the initiation of asthma. Intestinal and respiratory mucosa share similar bacterial communities and mucosal responses. The gut microbiome influences the respiratory microbiome as a source of bacterial colonization of the airways or by the metabolome of intestinal bacteria. The gut microbiome has also been shown to affect the development of atopy^23^. The contribution of gut dysbacteriosis in asthma is mostly acknowledged in early childhood and is less elucidated in adult asthmatics^24,25^. This might be due to the complicated intestinal microbiome of adults, requiring consideration of diverse host factors, such as living environment and diet. Recently, the distinct gut microbiota has also been revealed in adult asthmatics^26,27^. Supporting this phenomenon, microbe-derived short-chain fatty acids were suggested as possible mediators in the pathogenesis of asthma^28^. Our study demonstrated the alteration of overall body microbiota in established adult asthmatics by using blood EVs. However, we cannot assess causality between the examined taxa and asthma initiation. Further functional studies are required to understand the role of residential bacteria in the context of the development and progress of asthma in adults.

Numerous researchers have presented distinct respiratory microbiota according to asthma phenotypes or endotypes^29^. When we performed subgroup analysis, no difference was noted at the phylum level, whereas distinct changes of microbiota members were observed at the genus level. In eosinophilic asthma (defined by blood eosinophil counts), there was an abundance of *Escherichia/Shigella*. However, different results were obtained when the inflammatory type was decided based on the characteristics of induced sputum. Among the four subgroups, *Comamonas*, a genus of Proteobacteria and family *Comamonadaceae*, was notably high in the mixed granulocytic asthma. *Comamonas* was identified in the respiratory secretions of patients with cystic fibrosis and has been shown to have steroid degrading capacity^30–32^. When comparing severe asthmatics with mild-to-moderate asthmatics, *Streptococcus* was one of bacterium correlated with lower lung function^29^. However, in the current study, we observed relative abundance of *Streptococcus* in the serum of asthmatics with better lung function. Corticosteroids have been suggested as an important factor affecting the relative abundance of the taxa of asthmatics^14^. When we stratified the patients by steroid naïve, ICS only, or both ICS and OCS use, ICS tended to decrease the relative abundance of *Streptococcus*,* Lactobacillus*,* Staphylococcus*, and *Rothia*. By contrast, *Prevotella 9*,* Intestinibacter*, and *Blautia* were more abundant in patients using both ICS and OCS. However, since most of the patients were categorized in the ICS group, the effect of ICS with the addition of OCS might have been underestimated. Additionally, the treatment duration of corticosteroids was not considered, which might be necessary to address the effect of ICS and additional OCS.

EVs are released from various cell types, and function as intercellular communicators. As to the bacterial EVs, their immunomodulatory role has been persistently investigated in both gram-negative and -positive bacteria, although the latter remains poorly elucidated^33^. Outer membrane vesicles of *E. coli* and *P. aeruginosa* were reported to induce neutrophilic inflammation in the lung of mice^34,35^. Repeated exposure to *S. aureus* EVs also induced Th1 and Th17 neutrophilic pulmonary inflammation^36^. Although we could not decipher the contribution of pathogen specific EVs to the pathophysiology of asthma, distinct microbiota has raised questions on the role of molecules harbored by EVs other than DNA. However, it is uncertain whether the concentrations of EVs from colonized bacteria are sufficient to induce certain immunologic pathways, as their proinflammatory role has been proven when used in large amounts.

In this study, we used serum EVs to identify bacterial composition. Recently, metagenomic analyses of urine and serum bacterial-derived EVs identified distinct microbiota in patients with various allergic diseases^37,38^. To date, most studies regarding asthma microbiomes were conducted using specimens from various sections of the airways with different methods. Analyzing the sputum might be easy but poses issues of adequacy and contamination. Bronchoscopy can be more precise to directly investigate airways; however, it is technically demanding and invasive. In contrast, blood sampling is relatively quick and easy and does not require a standardized protocol. Additionally, blood EVs enabled comprehensive assessment of the microbiota of individuals and were not confined to a specific organ. Although evaluation of local changes in the microbiome must be determined, especially on the mucosal surface, overall microbiota differences between asthma patients and healthy controls appear to be evident.

There are some major limitations to our study. Firstly, as we analyzed the EVs only in blood samples, the origin and location of the bacteria remained unknown. Despite the similarities in dominant microbiota between blood and gastrointestinal tract, the composition may alter differently, depending on the medical conditions of the host^39^. As to blood microbiota, there are many sources other than gut, such as the respiratory tract or oral cavity. However currently, there is no feasible way of assessing the origin and immunomodulatory effects of microbiota when detected in circulating blood. To better understand the gut-lung axis in asthma using EVs, simultaneous metagenomic analyses of respiratory and gastrointestinal tract, as well as blood EVs in one individual, are warranted. Second, diverse host factors, including diet patterns, smoking status, and concomitant use of antibiotics, were not evaluated in this study, which might have affected the host microbiome. Third, since this was a cross-sectional study, it was challenging to conclude the role of identified bacteria in the progress and pathogenesis of asthma. Lastly, we did not perform density gradient centrifugation when collecting bacterial membrane vesicles. Even though we overcame the contamination issues by heating and using a column-based isolation kit, further replication of the experiments using conventional methods may reinforce our findings. Nevertheless, to our knowledge, this is the first study on the analysis of serum microbiota of asthmatics relative to healthy controls. Moreover, this study evaluated characteristics of microbiota using a larger number of adult asthmatics than that in other studies thus far^7,10^.

In conclusion, our study demonstrated distinct patterns of the blood microbiome of asthma patients and proposed a diagnostic model. Our findings suggest great potential of serum EVs as an indicator for microbiome-based asthma diagnosis. Further studies are needed to interpret the distinct patterns of bacterial compositions in the context of airway inflammation and asthma subtypes.

## Methods

### Subjects and serum sample collection

Patients with asthma and healthy controls were enrolled from two university hospitals. The healthy controls were subjects who visited the hospitals for regular health screening. After completion of the checkup, we selected healthy individuals who were confirmed to have no known diseases and normal laboratory test results. This study was approved by the Institutional Review Board of Asan Medical Center and Haeundae Paik Hospital (IRB No. 2012-0234 and IRB No. 129792-2015-064). All methods in this study were conducted following the approved guidelines, and written informed consent was obtained from all clinical subjects.

### EV isolation and DNA extraction from human serum samples

To isolate EVs from human serum, whole blood was collected into serum separator tubes that were centrifuged at 3,000 × *g* at 4 °C for 15 min. We used 100 μL of serum from each participant. The supernatant was transferred into a new tube and centrifuged again (10,000 ×  *g* at 4 °C for 1 min) to remove floating particles. Next, the isolated serum was diluted tenfold with sterile phosphate-buffered saline, and bacteria and foreign particles were eliminated from the mixture by 0.22 μm filtration.

To extract DNA from the EVs, serum mixtures containing EVs were boiled at 100 °C for 40 min. After centrifugation (13,000 × *g* at 4 °C for 30 min), DNA was extracted from the supernatant free of floating particles by using the PowerSoil DNA Isolation Kit (MO BIO, USA), according to the manufacturer’s instructions. Finally, the extracted DNA was quantified using the QiAxpert system (QIAGEN, Germany).

### Bacterial metagenomic analysis using EV DNA from human serum samples

Extracted EV DNA was amplified by PCR using 16S_V3_F on the V3–V4 hypervariable region in 16S rDNA (5′-TCGTCGGCAGCGTCAGATGTGTATAAGAGACAGCCTACGGGNGGCWGCAG-3′) and 16S_V4_R (5′-GTCTCGTGGGCTCGGAGATGTGTATAAGAGACAGGACTACHVGGGTATCTAATCC-3′) primers. The libraries were established with the amplified PCR products above as templates, according to the MiSeq system guide (Illumina, USA) and were quantified using QIAxpert (QIAGEN, Germany). Quantification, equimolar ratio setting, and pooling and sequencing on MiSeq for each amplicon were performed according to the manufacturer’s recommendations.

### Analysis of bacterial composition

We used cutadapt V1.1.6 to trim paired-end reads that were matched to the adapter sequences^40^. Next, we merged FASTAQ files containing paired-end reads with CASPER and did quality filter with Phred (Q) score^41,42^. Sequences shorter than 350 bp or longer than 550 bp were discarded. To identify chimeric sequences, we performed VSEARCH, including a reference-based chimera detection step against the SILVA gold database^43,44^. Subsequently, we clustered the sequence reads into operational taxonomic units (OTUs) using VSEARCH with a de novo clustering algorithm under a threshold of 97% sequence similarity. Finally, we classified the representative sequences of OTUs using the SILVA 128 database with UCLUST (parallel_assign_taxonomy_uclust.py script on QIIME version 1.9.1) under default parameters^45^.

### Statistical analysis

We performed statistical analyses using Chi-square tests for categorical variables and t-tests and Wilcoxon rank-sum tests for continuous variables. Then, we calculated species richness, evenness, and diversity indices and displayed as Chao1, Shannon, and Simpson index. The resultant distance matrix used for drawing an ordination diagram used the principal coordinates analysis (PCoA) based on Bray–Curtis distance.

### Development of a diagnostic model of asthma

For biomarker selection, we used stepwise selection and the linear discriminant analysis (LDA) effect size (LEfSe) method with consideration of statistical significance and biological relevance in the following four categories: (1) selected with a stepwise method, (2) incorporated with age and sex as covariates to the stepwise selection, (3) selected with the LEfSe algorithm, and (4) incorporated with age and sex for LEfSe selection. We selected biomarkers by LEfSe with an average abundance greater than 1% in any group, a p-value less than 0.05 by the Kruskal–Wallis rank-sum test between the control and asthmatics, and an LDA score greater than 3. After randomly dividing the samples into a training set and test set at an 80:20 ratio, logistic regression using biomarkers was performed to develop a diagnostic model following training and validation. All statistical analyses were performed using R version 3.4.1.

## Data Availability

The raw data supporting the conclusions of this manuscript will be made available by the authors, without undue reservation, to any qualified researcher upon request.

## Abbreviations

EV: Extracellular vesicle
AD: Alzheimer’s disease
OUT: Operational taxonomic units
LDA: Linear discriminant analysis
LefSe: Linear discriminant analysis effect Size
FEV1: Forced expiratory volume in 1 s
ICS: Inhaled corticosteroids
OCS: Oral corticosteroids
